# Successful treatment of nephrotic syndrome due to pregnancy-related crescentic IgA nephropathy: a case report

**DOI:** 10.1186/s12882-023-03152-y

**Published:** 2023-04-10

**Authors:** Hisato Shima, Toshio Doi, Takuya Okamoto, Tomoko Inoue, Manabu Tashiro, Seiichiro Wariishi, Kazuhiko Kawahara, Kazuyoshi Okada, Jun Minakuchi

**Affiliations:** 1 Department of Kidney Disease, Kawashima Hospital, 6-1 Kitasakoichiban-Cho, Tokushima, 770-0011 Japan; 2 Department of Laboratory, Kawashima Hospital, 6-1 Kitasakoichiban-Cho, Tokushima, 770-0011 Japan; 3 Department of Cardiovascular Surgery, Kawashima Hospital, 6-1 Kitasakoichiban-Cho, Tokushima, 770-0011 Japan

**Keywords:** Crescentic IgA nephropathy, Nephrotic syndrome, Pregnancy, Steroid, Normotensive

## Abstract

**Background:**

Crescentic immunoglobulin A (IgA) nephropathy, defined as > 50% of the glomeruli with crescents, often has a poor renal prognosis. Because of the high prevalence of pre-eclampsia in the second trimester of pregnancy, we often fail to investigate the new onset of glomerulonephritis and the aggravation of subclinical nephropathies. We report a case of nephrotic syndrome suggestive of crescentic IgA nephropathy possibly triggered by pregnancy.

**Case presentation:**

A 33-year-old multipara was referred for persistent proteinuria, hematuria, and hypoalbuminemia two months postpartum. The patient was diagnosed with proteinuria for the first time at 36 weeks of gestation. The patient was normotensive during pregnancy. Renal biopsy revealed crescentic IgA nephropathy, with cellular crescents in 80% of the glomeruli and no global sclerosis. After treatment with pulse steroids followed by high-dose oral glucocorticoids and tonsillectomy, a gradual improvement was seen in proteinuria, hematuria, and hypoalbuminemia.

**Conclusion:**

Although the precise mechanism remains unclear, pregnancy possibly triggered the new onset of crescentic IgA nephropathy or the aggravation of subclinical IgA nephropathy.

**Supplementary Information:**

The online version contains supplementary material available at 10.1186/s12882-023-03152-y.

## Background

Immunoglobulin A (IgA) nephropathy is the most common type of glomerulonephritis worldwide and is characterized by mesangial deposits of IgA1, often with co-deposits of Complement component 3 (C3) [1]. It is a common disease among young people and often occurs in women of childbearing age. Patients with IgA nephropathy rarely present with nephrotic syndrome (NS). Crescentic IgA nephropathy, defined as more than 50% of the glomeruli with crescents, is rare (1.14%) and sometimes presents with nephrotic syndrome [2, 3]. Its renal survival rate is only 50% at 1 year and 20% at 5 years [4]. However, the pathogenesis of crescent formation in IgA nephropathy remains unclear. Because of the high prevalence of pre-eclampsia in the second trimester of pregnancy, we often fail to investigate the new onset of glomerulonephritis and aggravation of subclinical nephropathies [5].

Herein, we report a case of nephrotic syndrome due to crescentic IgA nephropathy, possibly triggered by pregnancy. To the best of our knowledge, it has not been reported that pregnancy possibly triggered the new onset of crescentic IgA nephropathy or the aggravation of subclinical IgA nephropathy.

## Case report

**Fig. 1:**
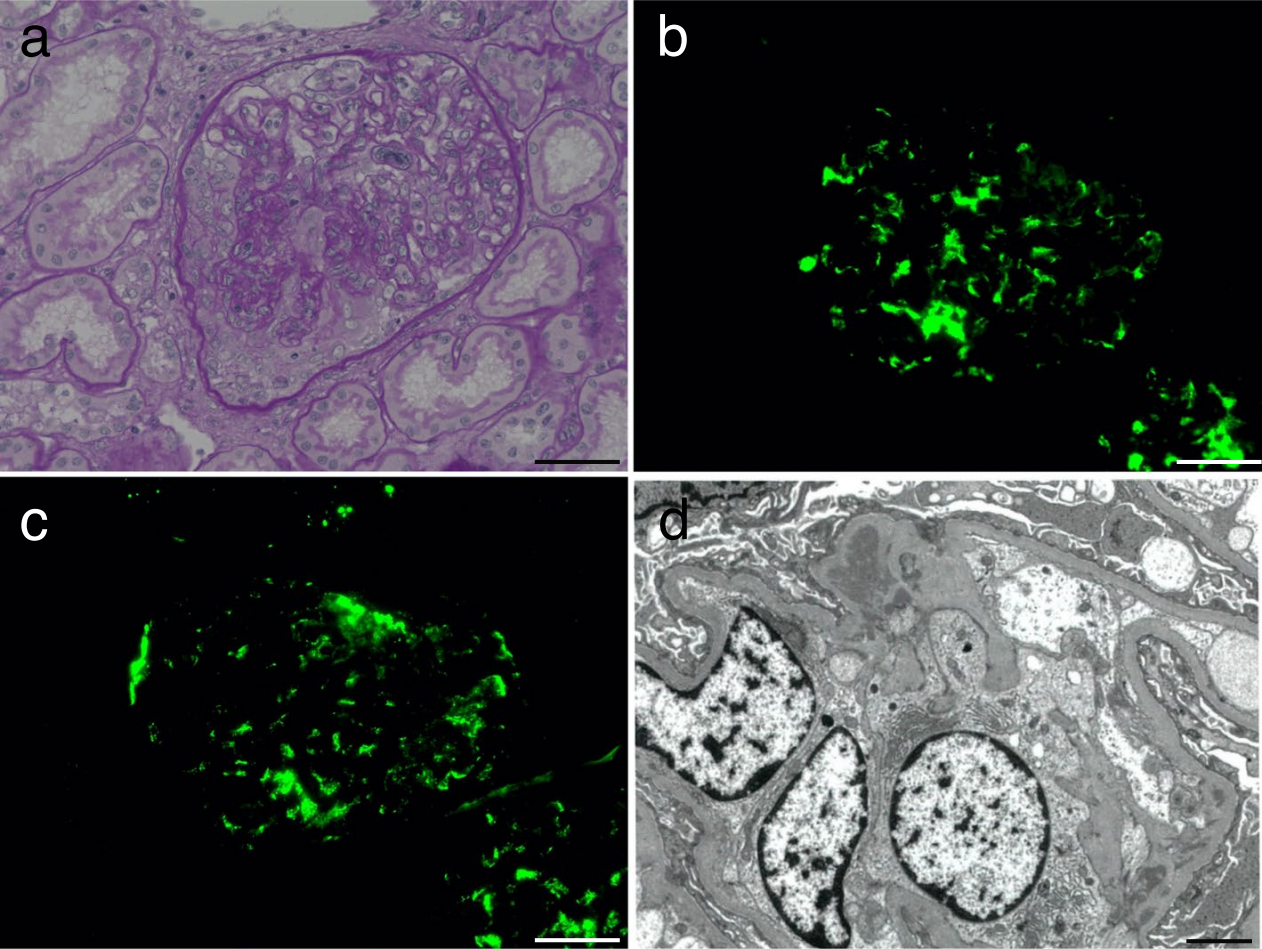
Renal biopsy specimen by light microscopy. **a** Glomerulus with a cellular crescent. Representative Periodic acid-Schiff staining (400 × magnification), Scale bars is 50 µm; Immunofluorescence staining shows a granular pattern for (**b**) IgA and (**c**) C3 in the mesangium (400 × magnification), Scale bars is 50 µm, Images were acquired using BZ-X710 all-in-one fluorescence microscope with BZ-X Viewer program (Keyence, Osaka, Japan). No enhancement of the images was performed. The measured resolution was 4080 × 3060; **d** Electron microscopy (Hitachi HT7700, Tokyo, Japan) shows electron-dense deposits mainly in mesangial area (4000 × magnification), Scale bars is 20 µm

**Fig. 2:**
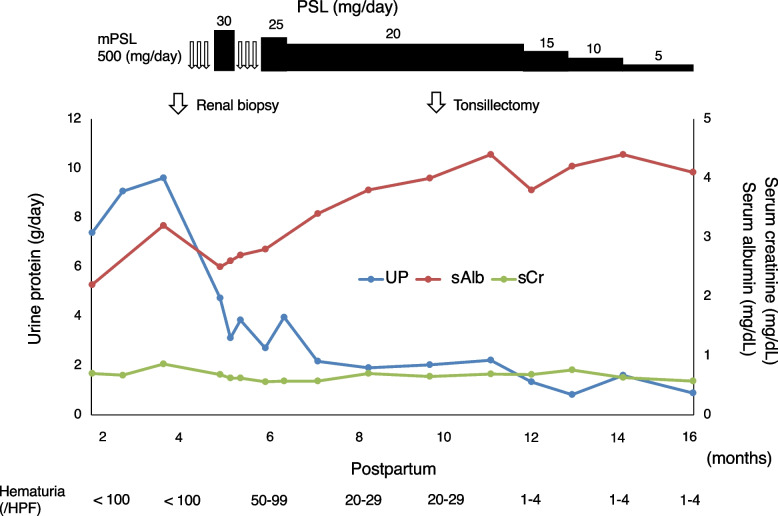
Clinical course of the patient. UP, urine protein; sAlb, serum albumin; sCr, serum creatinine; PSL, prednisolone; mPSL, methylprednisolone

A 33-year-old multipara was referred for persistent proteinuria, hematuria, and hypoalbuminemia two months postpartum. Her serum creatinine (sCr) level was 0.43 mg/dL, with no proteinuria and hematuria observed before pregnancy. She was diagnosed with proteinuria for the first time at 36 weeks of gestation. Her sCr levels were 0.45 mg/dL and 0.61 mg/dL at 37 and 38 weeks of gestation, respectively. There were no signs of infection immediately prior to the onset of proteinuria. She was normotensive with no symptoms such as swelling, headaches, upper abdominal pain, or shortness of breath before and after 36 weeks. Further, she showed no signs of cytomegalovirus or chlamydia infection during pregnancy. She had no significant medical history, allergies, or medications. There were no problems with her previous pregnancy. At 38 weeks, labor was induced, and she delivered a 3,290 g male infant. She presented with extensive peripheral edema on her first visit. She did not present with purpura, arthralgia, or abdominal pain. The laboratory findings on her first visit at two months postpartum are summarized in Table 1. Urinalysis revealed proteinuria (7.39 g/gCr) and hematuria (sediment red blood cells > 100 per high-power field). Urinary excretion of beta2-microglobulin (MG) and N-acetyl-beta-D-glycosaminidase were markedly elevated (1079 µg/mL and 90.1 U/L, respectively). Her sCr level was normal (0.70 mg/dL). She had low serum total protein and albumin levels of 5.3 g/dL and 2.2 g/dL, respectively. Based on these results, the patient was diagnosed with nephrotic syndrome. The antinuclear antibody titer was 1:160. She also tested negative for anti-DNA, IgG anticardiolipin antibodies, myeloperoxidase anti-neutrophil cytoplasmic, proteinase 3 anti-neutrophil cytoplasmic antibodies (ANCA), anti-glomerular basement membrane (anti-GBM), and anti-Smith antibodies. Serum protein electrophoresis revealed no monoclonal spikes. Renal ultrasound showed that the kidneys were normal in size (right, 108 × 51 mm; left, 110 × 55 mm) without dilation of the urinary tract, renal pelvis, or calyces. The corticomedullary junction was obscured. The renal arterial resistive index was normal (right, 0.50; left, 0.51). A renal biopsy was performed because the urinary protein persisted until 4 months postpartum. There were 35 glomeruli with no global sclerotic glomeruli. Diffuse and moderate mesangial proliferation and crescent formation were also observed. Crescent formation (28 cellular and one fibro-cellular) was observed in 29 of 35 glomeruli (Fig. 1a and Supplementary Fig). Endotheliosis was not observed in the glomeruli. No fibrinoid necrosis was observed in the glomeruli or arteries. IgA immunofluorescence staining showed a strong granular pattern for IgA (Fig. 1b) associated with IgG, IgM, and C3 (Fig. 1c) and fibrinogen levels in the mesangium. C1q and C4 levels were negative. Interstitial fibrosis and tubular atrophy were not observed. Electron microscopy showed electron-dense deposits, mainly in the mesangial area (Fig. 1d). Based on these findings, the patient was diagnosed with crescentic IgA nephropathy (M1E1S1T0C2 according to the Oxford Classification [6]). The clinical course of the patient is shown in Fig. 2. She was treated with methylprednisolone (mPSL) pulse therapy, followed by conventional prednisolone therapy. A tonsillectomy was performed 10 months postpartum. Both proteinuria and serum albumin levels gradually improved (0.89 g and 4.1 mg/dL, respectively), as well as hematuria at 16 months postpartum.

**Table 1:** Laboratory data

Blood								
WBC	6900	/μL	Triglyceride	200	mg/dL	PT-INR	0.97	
RBC	4.76 × 10^6^	/μL	LDL-cholesterol	321	mg/dL	APTT	30.0	sec
Hemoglobin	13.9	g/dL	HDL-cholesterol	71	mg/dL	aCL IgG	2	
Hematocrit	41.9	%	C-reactive protein	0.64	mg/dL	HBs Ag	−	
Platelet count	666 × 10^3^	/μL	ASO	27	IU/mL	HCV Ab	−	
Total protein	5.3	g/dL	IgG	472	mg/dL	T-SPOT	−	
Albumin	2.2	g/dL	IgA	371	mg/dL			
Total bilirubin	0.2	mg/dL	IgM	88	mg/dL	Urine		
BUN	12.2	mg/dL	IgE	45	IU/mL	Dipstick protein	3 +	
Creatinine	0.70	mg/dL	C3	136	mg/dL	Occult blood	3 +	
Uric acid	4.6	mg/dL	C4	29.3	mg/dL	RBC	> 100	/HPF
AST	27	mg/dL	CH50	58	mg/dL	Protein	7.39	g/gCr
ALT	20	IU/L	ANA	160		β_2_ MG	1079	µg/mL
LDH	260	IU/L	Anti-DNA ab	−		NAG	90.1	IU/L
ALP	77	IU/L	RF	6	IU/mL			
γGTP	12	IU/L	MPO-ANCA	−				
CK	83	IU/L	RP3-ANCA	−				
Sodium	141	mEq/L	Anti-GBM Ab	−				
Potassium	3.7	mEq/L	Anti-Sm Ab	−				
Chloride	106	mEq/L	TSH	1.02	µU/mL			
Calcium	8.3	mg/dL	FT3	2.0	pg/mL			
Phosphorus	3.3	mg/dL	FT4	0.98	ng/dL			

*WBC* White blood cell, *RBC* Red blood cell, *BUN* Blood urea nitrogen, *AST* Aspartate aminotransferase, *ALT* Alanine aminotransferase, *LDH* Lactate dehydrogenase, *ALP* Alkaline phosphatase, *γGTP* *γ* − glutamyl transpeptidase, *HDL* High density lipoprotein, *LDL* Low density lipoprotein, *Ig* Immunoglobulin, *C3* Complement component 3, *C4* Complement component 4, *CH50* 50% Hemolytic complement*, ANA* Antinuclear antibody, *RF* Rheumatoid factor, *MPO-ANCA* Myeloperoxidase anti-neutrophil cytoplasmic antibody, *PR3-ANCA* Proteinase-3 anti-neutrophil cytoplasmic antibody, *GBM* Glomerular basement membrane, *Sm* Smith, *PT-INR* prothrombin time-international normalized ratio, *APTT* Activated partial thromboplastin time, *aCL* Anticardiolipin antibodies, *HBs* Hepatitis B surface, *HCV* Hepatitis C virus, *HPF* High power field, *β*_*2*_* MG* Beta2-microglobulin, *NAG* N-acetyl-beta-D-glucosaminidase

## Discussion and conclusions

We report a rare case of nephrotic syndrome in a 33-year-old multipara that might be attributed to pregnancy-related crescentic IgA nephropathy. There has been insufficient data regarding the new onset of glomerulonephritis or aggravation of subclinical nephropathies in the course of gestation [5]. Although the precise mechanisms remain unclear, we assume that crescentic IgA nephropathy is related to pregnancy for two reasons. First, no urinary abnormalities were observed before 36 weeks of gestation. Second, renal biopsy revealed that most crescents were cellular, with no global sclerosis. These findings suggest a relatively new-onset crescentic IgA nephropathy during pregnancy. Excessive complement activation may be involved in crescent formation, and complement inhibition with the humanized anti-C5 monoclonal antibody eculizumab may be beneficial for crescentic IgA nephropathy [7, 8]. In general, complement activation may play a key role in placental formation and pregnancy maintenance [9]. It is possible that crescentic IgA nephropathy is related to pregnancy through complement activation. In this case, plasma C3, C4, and CH50 levels were normal. However, further complements, including factor H, factor B, C1q, mannose-binding lectin, C3c, C3a, C5a, and soluble C5b-9 have not been analyzed. Further studies are needed to assess whether pregnancy induces crescentic IgA nephropathy through complement activation. Crescent glomerulonephritis includes pauci-immune, immune-complex-mediated, and anti-glomerular basement membrane diseases. Macrophage inflammatory protein-1α may be involved in the development of cellular crescents in crescentic glomerulonephritis [10]. In this case, there was no fibrinoid necrosis in the glomeruli and small arteries, and ANCA and anti-GBM antibody data were negative. Therefore, ANCA-associated vasculitis and anti-GBM diseases are less likely. IgA nephropathy is an immune-complex-mediated glomerulonephritis that is recognized as an autoimmune renal disease due to increased circulating levels of IgA1 with galactose-deficient hinge region O-glycans and antiglycan autoantibodies [11]. There are some reports of Henoch-Schonlein purpura (HSP) during pregnancy [12, 13]. In this case, the possibility of HSP was low because of the absence of purpura, arthralgia, and abdominal pain. HSP and IgA nephropathy are considered related diseases resulting from the glomerular deposition of aberrantly glycosylated IgA1 [14]. There are a number of parameters other than the complement that could be altered due to pregnancy, including the gut microbiota [15], which might affect levels of IgA1 glycosylation, and the galactose-deficient IgA1 immune complex. Although the effects of pregnancy on the course of HSP and IgA nephropathy remain unclear, a similar mechanism may be involved, and further research is needed. Secondary forms of IgA nephropathy have been reported [16] and include gastrointestinal and liver disorders, infections, autoimmune disorders, and neoplasia [16]. Although we could not differentiate all diseases, our findings indicated that secondary IgA nephropathy was less likely. Pre-eclampsia is the most frequent renal complication of pregnancy and is characterized by hypertension and proteinuria after 20 weeks of gestation. Pre-eclampsia is characterized by glomerular capillary endotheliosis [17], and some patients do not have hypertension [18]. The rate of pre-existing renal disease in preeclamptic women was 71%, including IgA nephropathy (approximately 40%) [5]. It is difficult to distinguish between preexisting IgA nephropathy and preeclampsia during late pregnancy. In this report, the patient was normotensive before, during, or after pregnancy with no glomerular capillary endotheliosis. She had no organ symptoms, such as cerebrovascular events, hepatic failure, or HELLP syndrome (hemolysis, elevated liver enzymes, and low platelets) before or after 36 weeks. In addition, the presence of hematuria is not typical of preeclampsia [19]. Although the pathogenic role of many immunologic changes occurring during pregnancy remains unclear, we assume that pregnancy possibly triggered the new onset of crescentic IgA nephropathy or the aggravation of subclinical IgA nephropathy. In the present case, according to the International IgA nephropathy Prediction Tool [20], the risk of a 50% decline in estimated GFR or progression to end-stage renal disease 2 years after the landmark time post biopsy was 39.0%. Thus, it could be proposed that early diagnosis and treatment of crescentic IgA nephropathy, including pulse steroids followed by high-dose oral glucocorticoids and tonsillectomy, were crucial to achieve recovery from nephrotic syndrome and maintain renal function. In conclusion, this report describes an intriguing case in which pregnancy may trigger a new onset of crescentic IgA nephropathy or aggravation of subclinical IgA nephropathy.

## Supplementary Information

**Additional file 1: Supplementary Figure.** Representative renal histological images of Periodic acid-Schiff staining (400× magnification). Scale bars, 50 µm.

## Data Availability

All data generated and analyzed during this study were included in this published article.

## Abbreviations

IgA: Immunoglobulin A
C3: Complement component 3
NS: Nephrotic syndrome
sCr: Serum creatinine;
MG: Micro-globulin
IgG: Immunoglobulin G
ANCA: Anti-neutrophil cytoplasmic antibodies
GBM: Glomerular basement membrane
IgM: Immunoglobulin M
C4: Complement component 4
mPSL: Methylprednisolone
C5: Complement component 5
CH50: 50% Hemolytic complement
C1q: Complement component 1q
C3c: Complement component 3c
C3a: Complement component 3a
C5a: Complement component 5a
HSP: Henoch-Schonlein purpura
