# Supplementary material for: Successful treatment of nephrotic syndrome due to pregnancy-related crescentic IgA nephropathy: a case report
Source: BMC Nephrol. 2023 Apr 10;24:92. doi: 10.1186/s12882-023-03152-y (PMC10084611; doi:10.1186/s12882-023-03152-y)
Supplement: Supplementary file 1 — Additional file 1: Supplementary Figure. Representative renal histological images of Periodic acid-Schiff staining (400× magnification). Scale bars, 50 µm. [file 12882_2023_3152_MOESM1_ESM.pdf]

Supplementary Figure

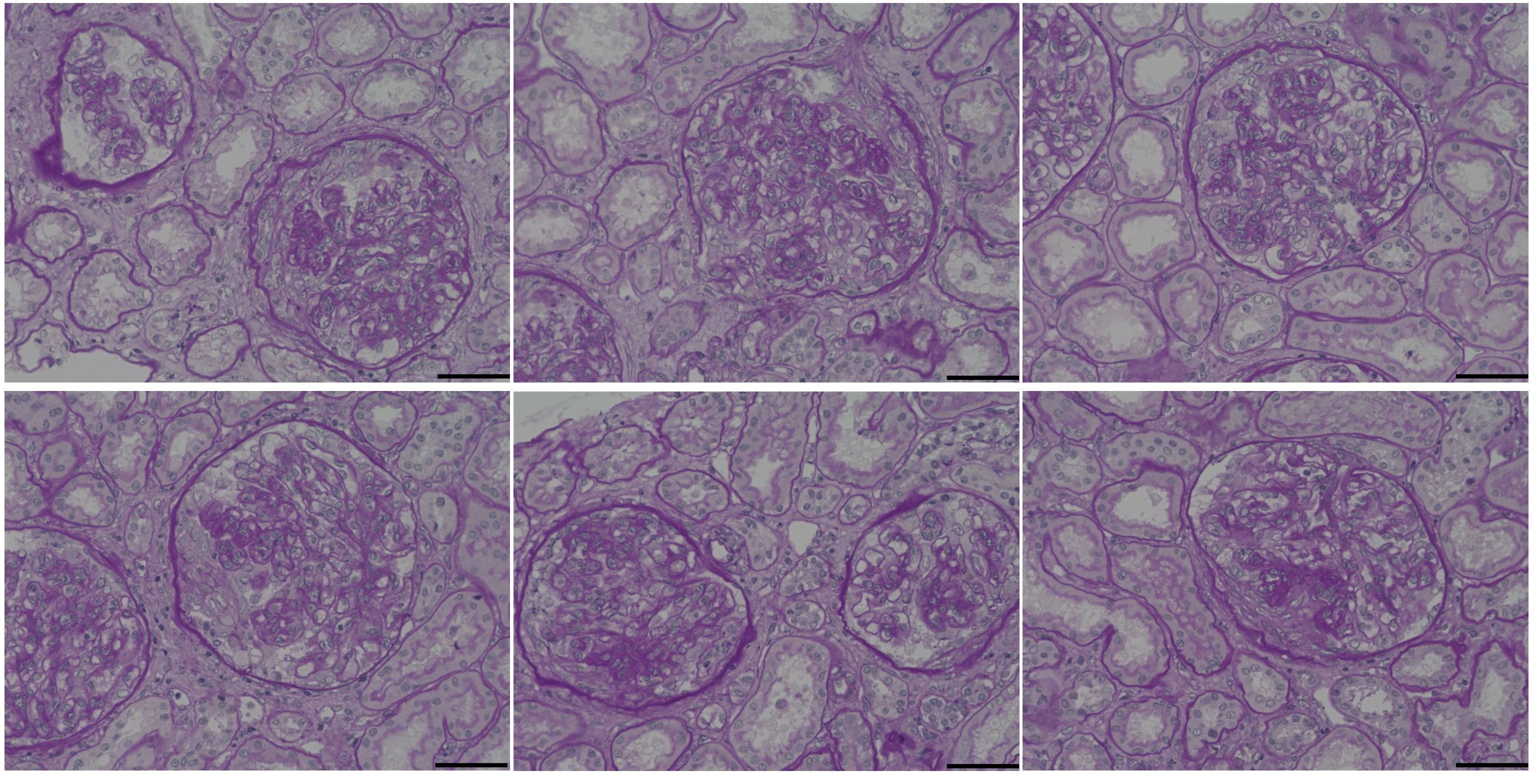

Supplementary Figure
Representative renal histological images of Periodic acid-Schiff staining (400× magnification).
Scale bars, 50 μm.
